# Balloon-expandable transcatheter aortic valve implantation with or without pre-dilation – results of a meta-analysis of 3 multicenter registries

**DOI:** 10.1186/s12872-019-1151-y

**Published:** 2019-07-19

**Authors:** Jannik Ole Ashauer, Nikolaos Bonaros, Markus Kofler, Gerhard Schymik, Christian Butter, Mauro Romano, Vinayak Bapat, Justus Strauch, Holger Schröfel, Andreas Busjahn, Cornelia Deutsch, Peter Bramlage, Jana Kurucova, Martin Thoenes, Stephan Baldus, Tanja K. Rudolph

**Affiliations:** 1 Department of Cardiology, University of Cologne Heart Center, Cologne, Germany; 2 Department of Cardiac Surgery, Medical University Innsbruck, Innsbruck, Austria; 3 Medical Clinic IV, Department of Cardiology, Municipal Hospital, Karlsruhe, Germany; 4 Department of Cardiology, Immanuel Clinic Bernau, Heart Center Brandenburg, Bernau, Germany; 5 Institut Hospitalier Jacques Cartier, Massy, France; 6 Columbia University Medical Center / New York-Presbyterian Hospital, New York, NY USA; 7 Clinic for Cardiosurgery and Thoracic Surgery, Berufsgenossenschaftliches Universitätsklinikum Bergmannsheil, Bochum, Germany; 8 Department Cardiovascular Surgery, University Heart Center Freiburg, Bad Krozingen, Germany; 9 Healthtwist GmbH, Berlin, Germany; 10 Institute for Pharmacology and Preventive Medicine, Bahnhofstrasse 20, 49661 Cloppenburg, Germany; 11 Edwards Lifesciences, Medical Affairs/Professional Education, Prague, Czech Republic; 12 Edwards Lifesciences, Medical Affairs/Professional Education, Nyon, Switzerland; 13 Department of Cardiology, Heart and Diabetes Center Bad Oeynhausen, Ruhr-University of Bochum, Bad Oeynhausen, Germany

**Keywords:** Transcatheter aortic valve implantation, Balloon aortic valvuloplasty, Pre-dilation, Aortic stenosis

## Abstract

**Background:**

To evaluate the outcomes of transcatheter aortic valve implantation (TAVI) without balloon aortic valvuloplasty (BAV) in a real-world setting through a patient-level meta-analysis.

**Methods:**

The meta-analysis included patients of three European multicenter, prospective, observational registry studies that compared outcomes after Edwards SAPIEN 3 or XT TAVI with (*n* = 339) or without (*n* = 355) BAV. Unadjusted and adjusted pooled odds ratios (with 95% confidence intervals) were calculated for procedural and 30-day outcomes.

**Results:**

Median procedural time was shorter in the non-BAV group than in the BAV group (73 versus 93 min, *p* = 0.001), as was median fluoroscopy time (7 versus 11 min, *p* = 0.001). Post-delivery balloon dilation (15.5% versus 22.4%, *p* = 0.02) and catecholamine use (9.0% vs. 17.9%; *p* = 0.016) was required less often in the non-BAV group than in the BAV group with the difference becoming insignificant after multiple adjustment. There was a reduced risk for periprocedural atrioventricular block during the intervention (1.4% versus 4.1%, *p* = 0.035) which was non-significant after adjustment. The rate of moderate/severe paravalvular regurgitation post-TAVI was 0.6% in the no-BAV group versus 2.7% in the BAV group. There were no between-group differences in the risk of death, stroke or other adverse clinical outcomes at day 30.

**Conclusions:**

This patient-level meta-analysis of real-world data indicates that TAVI performed without BAV is advantageous as it has an adequate device success rate, reduced procedure time and no adverse effects on short-term clinical outcomes.

## Background

The conventional approach for transcatheter aortic valve implantation (TAVI) includes pre-dilation balloon aortic valvuloplasty (BAV) to help estimate prosthetic valve size, facilitate delivery of the TAVI catheter across the valve, optimize positioning and expansion of the prosthetic valve [1–4]. BAV can also be associated with adverse effects, however, such as hemodynamic instability, arrhythmia necessitating permanent pacemaker implantation, embolic events and stroke [1, 4–8]. Consequently, TAVI is increasingly being performed without BAV [9, 10]. Clinical studies of TAVI without BAV (direct TAVI) have provided encouraging pivotal results [11, 12]. Several subanalyses of larger registry studies later also suggested that direct TAVI is associated with good procedural results and clinical outcomes [9, 10, 13, 14]. To explore this topic further for balloon expandable valves is important for the following reasons: 1) Penetration of the calcified aortic valves is more cumbersome with balloon-expandable than with self-expanding valves due to the balloon and the annular skirt; 2) After successful implantation there is decreased need for post-implant dilatation (circular shape of the valve, lower rates of PV-leaks); 3) A limited balloon inflation can facilitate smooth introduction of the valve into the annulus.

To address this lack of data, three prospective, multicenter, registry studies evaluated TAVI using the balloon-expandable Edwards SAPIEN prosthetic valves, with and without BAV, and found that direct TAVI was feasible, safe and provided adequate efficacy in a real-world setting [15–17] (Schymik G, Rudolph TK, Jacobshagen C, Rothe J, Treede H, Kerber S, Frank D, Sykorova L, Okamoto M, Thoenes M, et al. Balloon-expandable transfemoral transcatheter aortic valve implantation with or without pre-dilation – findings from the EASE-IT TF multicentre registry, submitted). A meta-analysis of these three studies has now been conducted to provide additional information on the use of TAVI without BAV in a real-world setting.

## Methods

### Study characteristics

All three studies included in the meta-analysis were multicenter, prospective, observational registry studies conducted under the guidance of the *Institute for Pharmacology and Preventive Medicine* (Cloppenburg, Germany). Full details of the design and methodology for each study have been reported previously [18–20]. EASE-IT TF recruited patients undergoing transfemoral (TF) TAVI from 10 sites in Germany [19], EASE-IT TA recruited patients undergoing transapical (TA) TAVI from 10 sites in Germany as well [20], and ROUTE enrolled patients undergoing transaortic (TAo) TAVI from 18 sites across Europe [18]. Edwards SAPIEN 3 [18–20] or XT [18] transcatheter prosthetic heart valves were used in all studies. Patients were aged ≥18 years and had an indication for TAVI as evaluated by the center-specific heart team. Decisions about whether or not to perform BAV pre-dilation were made at the discretion of the treating physicians and were independent of inclusion in the registry. The individual studies enrolled between 196 and 300 evaluable patients [15–17] (Schymik G, Rudolph TK, Jacobshagen C, Rothe J, Treede H, Kerber S, Frank D, Sykorova L, Okamoto M, Thoenes M, et al. Balloon-expandable transfemoral transcatheter aortic valve implantation with or without pre-dilation – findings from the EASE-IT TF multicentre registry, submitted).

All three studies assessed outcomes at the time of the procedure and after 30 days, with the primary endpoints being composite safety endpoints (using definitions based on the Valve Academic Research Consortium-2 consensus document) at 30 days [21]. EASE-IT TF and EASE-IT TA had a 6 months follow-up, while ROUTE had no 6 months, but a 1 year follow-up.

### Data extraction

For the meta-analysis, data on patient and disease characteristics, procedural details and outcomes, and longer-term outcomes were extracted. Procedural details and outcomes included: device success, post-delivery balloon dilation, procedural time, fluoroscopy time, contrast agent volume, hemodynamic instability and inotropic support (both were not available for ROUTE), transvalvular pressure gradient and periprocedural complications. Longer-term outcomes included: mortality, stroke, non-fatal myocardial infarction, new-onset dialysis, acute kidney failure, permanent pacemaker implantation, life-threatening bleeding, major vascular complications, hospitalization, valve dysfunction, New York Heart Association (NYHA) class III or IV, and Canadian Cardiovascular Society Grading of Angina Pectoris class III or IV.

### Statistical analysis

Pooled data were compared between the group who underwent TAVI with BAV and the group who underwent TAVI without BAV. Unadjusted and adjusted pooled odds ratios (with 95% confidence intervals) were calculated for procedural outcomes and longer-term outcomes. Components of the composite safety endpoints used in the original studies were analyzed individually in the meta-analysis. Procedural and 30-day outcomes were also presented according to the access route used (TF, TA or TAo) with odds ratios (and 95% CI) for patients who did or did not undergo BAV. All statistical analyses were carried out using R version 3.4.3 (2017-11-30) [22], with a *p*-value of < 0.05 considered significant.

## Results

The pooled analysis population (*n* = 694) comprised 339 patients who underwent TAVI with BAV (including 56 TF, 61 TA and 222 TAo) and 355 who underwent TAVI without BAV (including 140 TF, 137 TA and 78 TAo).

### Baseline patient details

Baseline patient characteristics are summarized in Table 1. Those in the no-BAV group had a higher median bodyweight (75 versus 72 kg, *p* = 0.027) and body surface area (1.84 versus 1.81 cm^2^, *p* = 0.032) than those in the BAV group. A large proportion of patients in both groups had coronary artery disease and/or had undergone a prior cardiovascular intervention. Patients in the no-BAV group were less likely to have peripheral artery disease (23.9% versus 43.4%, *p* = 0.001) and more likely to have had a prior cardiovascular intervention (44.8% versus 36.3%, *p* = 0.025) compared with the BAV group, and had a higher median EuroSCORE II score (5 versus 4, *p* = 0.005). Aortic valve disease characteristics were similar in both groups.

**Table 1:** Baseline patient and disease characteristics

	N	TAVI with BAV *N* = 339	TAVI without BAV *N* = 355	*P*-value
Patient characteristics				
Age (years)	694	82 (79–86)	81 (78–86)	0.057
Female (%)	694	49.9	45.4	0.254
Height (cm)	694	166 (160–172)	168 (160–175)	0.052
Weight (kg)	694	72 (63–82)	75 (63–87)	0.027
Body surface area (cm^2^)^a^	694	1.81 (1.68–1.94)	1.84 (1.68–2.01)	0.032
BMI (kg/m^2^)	692	26 (23–29)	26 (24–30)	0.207
Hypertension (%)	678	85.2	88.4	0.255
Diabetes (%)	676	31.0	32.9	0.621
Stroke, TIA (%)	678	13.5	16	0.387
Peripheral artery disease (%)	679	43.4	23.9	< 0.001
Pulmonary hypertension (%)	527	33.0	38.6	0.202
Creatinine > 2 mg/dL (%)	694	5.9	6.2	0.875
Dialysis (%)	361	6.4	2.7	0.108
Coronary artery disease (%)	694	61.9	64.8	0.478
Prior myocardial infarction (%)	557	29.8	24.4	0.182
Prior CV intervention (%)	694	36.3	44.8	0.025
Prior pacemaker / ICD implant (%)	501	17.6	12.7	0.152
EuroSCORE II	588	4 (2–8)	5 (3–10)	0.005
STS Risk Score	616	5.3 (3.2–10.0)	4.6 (3.0–8.0)	0.002
Disease characteristics				
Echo AV peak PG (mmHg)	533	70 (57–82)	69 (55–81)	0.275
Echo AV mean PG (mmHg)	649	43 (35–54)	41 (33–50)	0.111
Echo V_max_ (m/s)	435	4.0 (3.7–4.4)	4.1 (3.6–4.5)	0.965
Echo ejection fraction (%)	653	55 (47–60)	55 (45–60)	0.324
Effective orifice area	534	0.70 (0.57–0.80)	0.70 (0.60–0.80)	0.043
Indexed effective orifice area^b^	533	0.38 (0.31–0.46)	0.39 (0.32–0.46)	0.348
NYHA Class III or IV	685	79.8	75.9	0.232
CCS grading of angina pectoris Class III or IV	645	16.8	17.0	1.000
Dizziness or syncope	694	30.1	30.1	1.000

Values are median (interquartile range) unless indicated otherwise

*AV* Aortic valve, *BAV* Balloon aortic valvuloplasty, *BMI* Body mass index, *CCS* Canadian cardiovascular society, *CV* Cardiovascular, *EuroSCORE* European system for cardiac operative risk evaluation, *ICD* Implantable cardioverter defibrillator, *NYHA* New York Heart Association, *PG* Pressure gradient, *STS* Society of thoracic surgeons, *TAVI* Transcatheter aortic valve implantation, *TIA* Transient ischemic attack, *V*_*max*_ Maximum velocity

^a^BSA [cm x kg] = 0.007184 x height [cm]^0.725^ x weight [kg]^0.425^ (DuBois, 1916)

^b^*iEOA* Effective orifice area/body surface area

### Periprocedural details

**Fig. 1:**
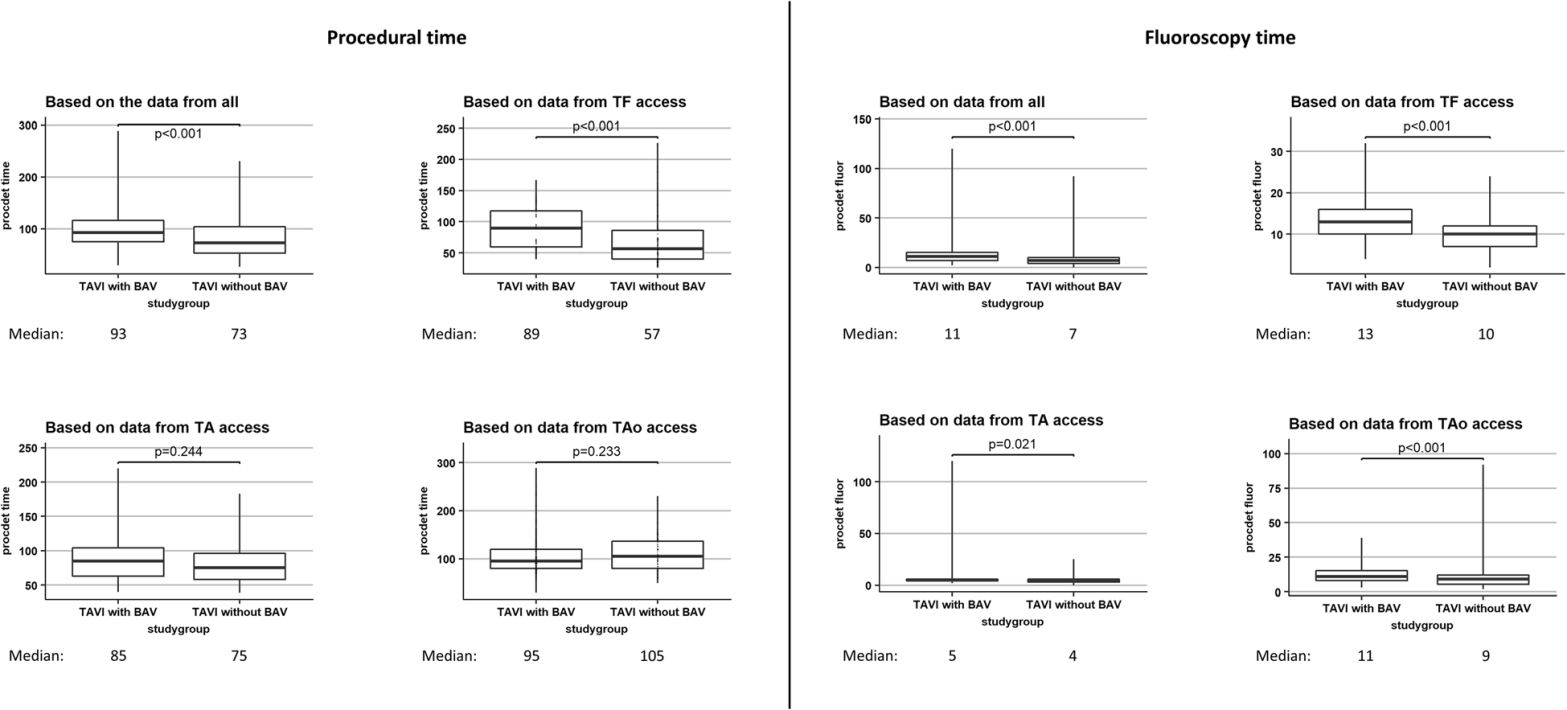
Procedural time/fluoroscopy time overall and by access route

The most commonly used valve size in both groups was 26 mm (Table 2). Post-delivery balloon dilation was required less often in the no-BAV group than in the BAV group (15.5% versus 22.4%, *p* = 0.02). Although the unadjusted odds ratio supported a reduced risk in the no-BAV group (OR 0.63, 95% CI 0.43–0.93), this was no longer significant in the adjusted analysis (aOR 0.67, 95% CI 0.41–1.06) (Table 3). Median total procedural time was significantly shorter in the no-BAV group compared with the BAV group (73 versus 93 min, *p* < 0.001; Fig. 1), as was the median fluoroscopy time (7 versus 11 min, *p* < 0.001; Fig. 1), and fewer patients in the no-BAV group received catecholamines (9.0% versus 17.9%, *p* = 0.016; aOR 0.56; 95% CI 0.24–1.38) (Tables 2 and 3).

**Table 2:** Procedural data and outcomes

	TAVI with BAV		TAVI without BAV		*P*-value
	N	Value	N	Value	
Valve size	339		355		< 0.001
20–23 mm		28.9		31.0	
26 mm		45.1		41.1	
29 mm		26.0		27.9	
Post-delivery balloon dilation (%)	339	22.4	355	15.5	0.020
Quantity contrast agent used (mL)	303	100 (73–131)	349	95 (70–126)	0.343
Access complications (%)	339	0.9	355	2.3	0.224
Hemodynamic instability (%)	117	5.1	277	2.5	0.219
Catecholamine use (inotropes) (%)	117	17.9	277	9.0	0.016
Effective orifice area post-surgery	119	1.89 (1.59/2.30)	114	1.80 (1.50/2.20)	0.378
Indexed effective orifice area	282	0.38 (0.31/0.46)	251	0.39 (0.31/0.46)	0.348
AV mean PG post-surgery	286	9.0 (6.0/12.0)	252	9.0 (6.0/12.8)	0.862
Paravalvular regurgitation	339		351		< 0.001
None/trace		78.5		84.3	
Mild		18.9		15.1	
Moderate		2.4		0.6	
Severe		0.3		0.0	
Device success (%)	339	97.9	355	99.2	0.214
Second valve needed (%)	339	1.2	355	0.8	0.719
Conversion to surgery (%)	141	3.5	280	2.1	0.518
Coronary artery obstruction requiring intervention (%)	213	0.9	71	0.0	1.000
Device malfunction (%)	339	0.6	355	0.3	0.616
Atrioventricular block (%)	339	4.1	355	1.4	0.035
Aortic root rupture (%)	339	0.3	355	0.0	0.488
Correct positioning of a single prosthetic valve into the proper anatomical location	339	99.1	355	99.7	0.363
Intended performance of the prosthetic valve	287	97.9	217	98.6	0.738

Values are median (interquartile range) unless indicated otherwise

*AV* Aortic valve, *BAV* Balloon aortic valvuloplasty, *CI* Confidence interval, *OR* Odds ratio, *PG* Pressure gradient, *TAVI* Transcatheter aortic valve implantation

**Table 3:** Procedural data and outcomes

	TAVI with BAV	TAVI without BAV	OR (95% CI)	Adjusted OR^a^ (95% CI)
Post-delivery balloon dilation (%)	22.4	15.5	0.63 (0.43–0.93)	0.67 (0.41–1.06)
Catecholamine use (Use of inotropes) (%)	17.9	9.0	0.45 (0.24–0.85)	0.56 (0.24–1.38)
Atrioventricular block (%)	4.1	1.4	0.33 (0.11–0.88)	0.44 (0.12–1.38)
Correct positioning of a single prosthetic valve into the proper location (%)	99.1	99.7	3.16 (0.4–64.07)	2.13 (0.24–45.99)
Intended performance of the prosthetic valve (%)	97.9	98.6	2.11 (0.55–10.08)	1.06 (0.22–5.69)

*BAV* Balloon aortic valvuloplasty, *CI* Confidence interval, *OR* Odds ratio, *TAVI* Transcatheter aortic valve implantation

^a^data were adjusted for age, gender, prior MI, stroke / TIA, creatinine, ejection fraction and NYHA class

### Procedural efficacy

The device success rate was high and did not differ significantly between groups (Table 2). The rate of device malfunctions was 0.3% in the no-BAV group versus 0.6% in the BAV groups (*p* = 0.616), although the adjusted odds ratio of 0.02 (95% CI 0.01–0.74) suggested the risk was reduced in the no-BAV group. Mean pressure gradients decreased from 41 to 9 mmHg in the no-BAV group and from 43 to 9 mmHg in the BAV group. Among procedural complications, atrioventricular block was significantly less common in the no-BAV group than the BAV group (1.4% versus 4.1%, *p* = 0.035); however, although the unadjusted odds ratio supported a reduced risk in the no-BAV group (OR 0.33, 95% CI 0.11–0.88), this was no longer significant in the adjusted analysis (aOR 0.44, 95% CI 0.12–1.38) (Table 3). Most patients had no paravalvular leakage after the procedure (no-BAV 84.3%; BAV 78.5%); moderate/severe paravalvular regurgitation was less common in the no-BAV group than the BAV group (*p* = 0.001; Table 2). There were no significant differences between groups for other procedural complications.

### 30-day outcomes

**Fig. 2:**
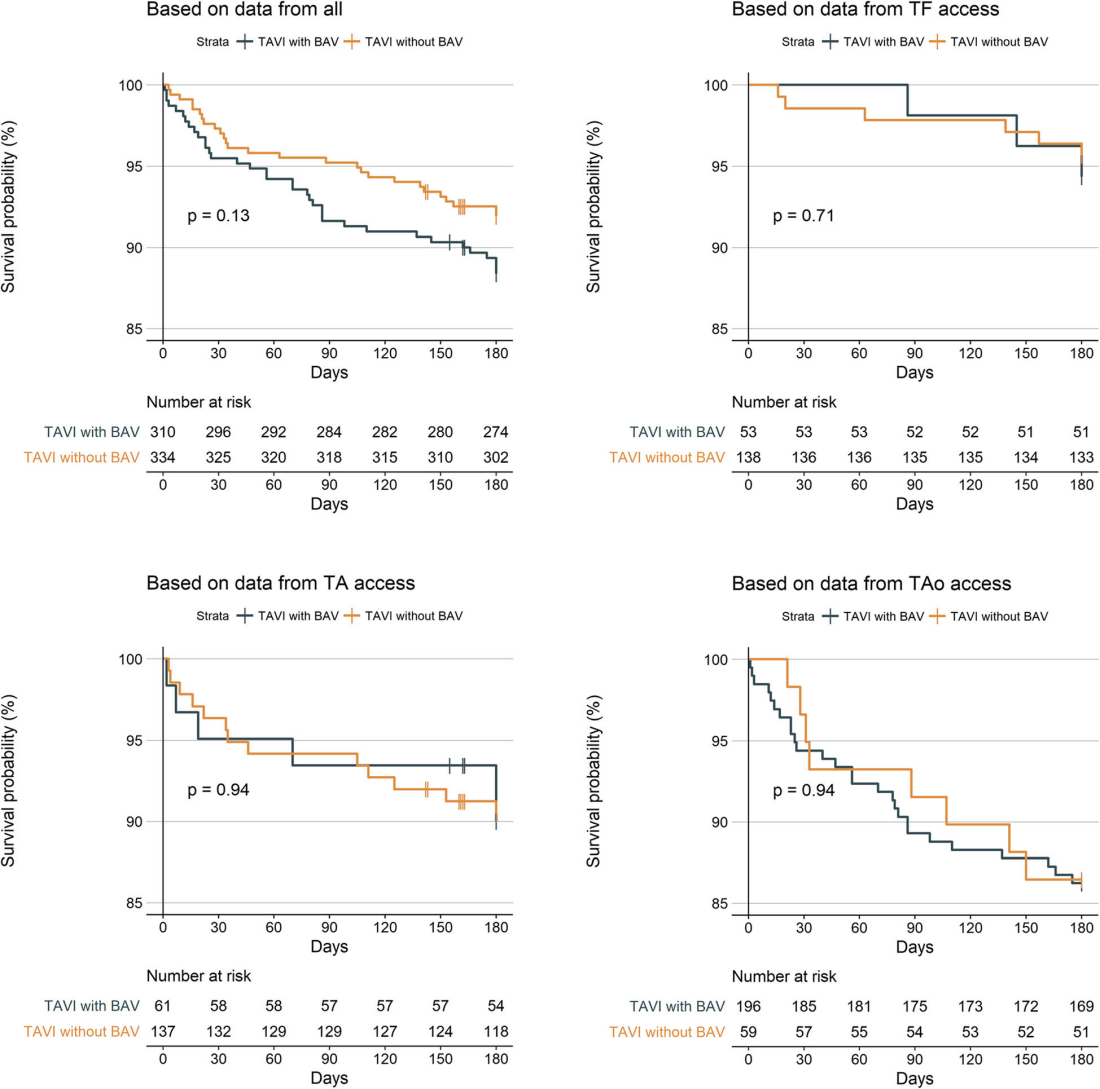
Survival probability overall and by access route. TAVI = transcatheter aortic valve implantation; BAV = balloon aortic valvuloplasty; TF = transfemoral; TA = transapical; TAo = transaortic; the X-axis is censored at 85% to illustrate the slight difference which is, however, not statistically significant even for the pooled cohort (*n* = 0.13); survival is illustrated up to the 6-month follow-up which has been captured in all three registries (EASE-IT TF 6 months, EASE-IT TA 6 months, ROUTE 1 year)

There were no significant between-group differences in the rate of death (Fig. 2), stroke, permanent pacemaker implantation or other outcomes at day 30 (Table 4), with the exception of patients in the no-BAV group being less likely in NYHA class III/IV than those in the BAV group (17.6% versus 54.5%; aOR 0.18, 95% CI 0.12–0.27).

**Table 4:** Outcomes at 30 days

	TAVI with BAV		TAVI without BAV		OR (95% CI)	
	N	%	N	%	Not-adjusted	Adjusted^a^
Death (%)	327	1.8	343	1.5	0.79 (0.23–2.65)	0.4 (0.06–1.86)
Stroke (%)	327	0.9	341	0.3	0.32 (0.02–2.5)	0.79 (0.02–27.7)
Non-fatal MI (%)	336	0.6	349	0.9	1.45 (0.24–11.05)	1.29 (0.12–13.1)
New-onset dialysis (%)	332	3.9	348	3.7	0.95 (0.43–2.1)	0.97 (0.4–2.32)
Creatinine increase (%)	268	1.5	210	1.4	0.96 (0.19–4.38)	0.42 (0.02–3.22)
Permanent pacemaker implantation (%)	337	10.1	350	8.6	0.84 (0.5–1.4)	1.17 (0.62–2.2)
Life-threatening bleeding (%)	333	3.0	345	1.4	0.48 (0.15–1.35)	0.42 (0.09–1.48)
Major vascular complications (%)	333	4.5	345	3.5	0.76 (0.35–1.66)	0.63 (0.21–1.65)
Hospitalization (%)	333	2.7	344	1.2	0.42 (0.11–1.31)	0.48 (0.09–1.9)
Valve dysfunction (%)	322	0.9	337	0.0	–	–
NYHA Class III or IV (%)	336	54.5	346	17.6	0.18 (0.13–0.25)	0.18 (0.12–0.27)
CCS grading of angina pectoris Class III or IV (%)	308	1.6	296	1.0	0.62 (0.13–2.55)	0.98 (0.18–4.8)

*BAV* Balloon aortic valvuloplasty, *CI* Confidence interval, *CCS* Canadian cardiovascular society, *MI* Myocardial infarction, *NYHA* New York Heart Association, *OR* Odds ratio, *TAVI* Transcatheter aortic valve implantation

^a^data were adjusted for age, gender, prior MI, stroke / TIA, creatinine, ejection fraction and NYHA class

### Outcomes by access route

Outcomes for patients receiving TAVI with or without BAV according to the access route are summarized in Table 5 (procedural outcomes) and Table 6 (30-day outcomes). The need for post-delivery balloon dilation was reduced in the no-BAV group compared with the BAV group among TF-TAVI patients (OR 0.43, 95% CI 0.21–0.89) but not TA-TAVI or TAo-TAVI patients (Table 5). Total procedure time was reduced in the no-BAV group compared with the BAV only among TF-TAVI patients (56.5 versus 89.5 min, *p* < 0.001) and not among TA-TAVI or TAo-TAVI patients (Fig. 1), whereas fluoroscopy time was reduced in the no-BAV group for all access routes (Fig. 1). The quantity of contrast agent used was reduced in the no-BAV group only among patients undergoing TAo-TAVI (80 versus 94 mL, *p* = 0.008). Use of inotropes was reduced in the no-BAV group only among patients undergoing TA-TAVI (17.5% versus 32.8%; OR 0.44, 95% CI 0.22–0.87). Most patients had no paravalvular regurgitation after TAVI irrespective of the use of BAV and route of access (76.6–86.0%). Moderate/severe paravalvular regurgitation appeared to be more common in the BAV group than the no-BAV group among those who underwent TAo-TAVI (Table 5).

**Table 5:** Procedural outcomes by access route

	TF access		TA access		TAo access	
	X_w_/X_wo_	*p*-value	X_w_/X_wo_	*p*-value	X_w_/X_wo_	*p*-value
Valve size		0.038		0.005		0.001
20–23 mm	37.5 / 31.4		29.5 / 30.7		26.6 / 30.8	
26 mm	23.2 / 39.3		49.2 / 40.9		49.5 / 44.9	
29 mm	39.3 / 29.3		21.3 / 28.5		23.9 / 24.4	
Quantity contrast agent used (mL)	131 / 120	0.240	85 / 80	0.681	94 / 80	0.008
Effective orifice area post-surgery	1.40 / 1.65	0.203	2.10 / 2.00	0.222	1.80 / 1.82	0.809
Indexed effective orifice area	0.71 / 0.87	0.175	1.17 / 1.04	0.137	1.04 / 1.00	0.857
AV mean PG post-surgery	12 / 11	0.138	4 / 5	0.641	9 / 10	0.863
Paravalvular regurgitation		0.001		< 0.001		< 0.001
None/trace	85.7 / 86.0		78.7 / 84.7		76.6 / 80.8	
Mild	14.3 / 13.2		21.3 / 14.6		19.4 / 19.2	
Moderate	0 / 0.7		0 / 0.7		3.6 / 0	
Severe	0 / 0		0 / 0		0.5 / 0	
	TF access		TA access		TAo access	
	X_w_/X_wo_	OR (95% CI)	X_w_/X_wo_	OR (95% CI)	X_w_/X_wo_	OR (95%CI)
Post-delivery balloon dilation (%)	30.4 / 15.7	0.43 (0.21–0.89)	14.8 / 9.5	1.65 (0.65–4.07)	22.5 / 25.6	0.84 (0.47–1.56)
Access complications (%)	0 / 5	n.a.	0 / 0.7	n.a.	1.4 / 0	n.a.
Hemodynamic instability (%)	3.6 / 0.7	0.19 (0.01–2.07)	6.6 / 4.4	0.65 (0.18–2.63)	n.c.	n.a.
Catecholamine use (%)	1.8 / 0.7	0.4 (0.02–10.12)	32.8 / 17.5	0.44 (0.22–0.87)	n.c.	n.a.
Device success (%)	92.9 / 98.6	5.31 (1.01–39.12)	100 / 100	n.a.	98.6 / 98.7	0.95 (0.05–7.53)
Second valve needed (%)	0 / 0.7	n.a.	0 / 0	n.a.	1.8 / 2.6	0.7 (0.13–5.1)
Conversion to surgery (%)	0 / 2.9	n.a.	0 / 1.5	n.a.	1.7 / 0	n.a.
Device malfunction (%)	0 / 0	n.a.	0 / 0.7	n.a.	0.9 / 0	n.a.
Atrioventricular block (%)	3.6 / 2.1	0.59 (0.1–4.58)	1.6 / 1.5	0.89 (0.08–19.34)	5 / 0	n.a.
Aortic root rupture (%)	0 / 0	n.a.	0 / 0	n.a.	0.5 / 0.0	n.a.
Correct positioning of a single prosthetic valve into the proper anatomical location (%)	100 / 100	n.a.	100 / 100	n.a.	98.6 / 98.7	1.05(0.13–21.5)
Intended performance of the prosthetic valve (%)	92.9 / 98.6	5.31 (1.01–39.1)	100 / 100	n.a.	99.1 / 98.7	0.7 (0.07–15.2)

Values are median (interquartile range) unless indicated otherwise

*AV* Aortic valve, *CI* Confidence interval, *n.a.* Not applicable (e.g. no ratio), *n.c.* Data not collected, *OR* Odds ratio, *PG* Pressure gradient, *TA* Transapical, *TAo* Transaortic, *TF* Transfemoral, *X*_*w*_ Transcatheter aortic valve implantation with balloon aortic valvuloplasty, *X*_*wo*_ *= TAVI* Transcatheter aortic valve implantation without balloon aortic valvuloplasty

**Table 6:** Outcomes at 30 days by access route

	TF access		TA access		TAo access	
	X_w_/X_wo_	OR (95%CI)	X_w_/X_wo_	OR (95%CI)	X_w_/X_wo_	OR (95%CI)
Death (%)	0 / 1.4	n.a.	0 / 0.8	n.a.	2.8 / 2.8	1 (0.14–4.46)
Stroke (%)	0 / 0	n.a.	0 / 0	n.a.	1.4 / 1.4	1 (0.05–7.95)
Non-fatal MI (%)	0 / 0.7	n.a.	1.6 / 0.7	0.44 (0.05–11.28)	0.5 / 1.4	2.99 (0.12–76.16)
New onset dialysis (%)	0 / 0.7	n.a.	4.9 / 5.1	1.04 (0.25–4.96)	9.2 / 10.3	1.13 (0.23–4.66)
Creatinine increase (%)	0 / 1.4	n.a.		–	1.9 / 1.4	0.74 (0.04–5.13)
PPI (%)	8.9 / 7.2	0.79 (0.27–2.64)	14.8 / 10.2	0.66 (0.27–1.67)	9.1 / 8.1	0.88 (0.31–2.17)
Life-threatening bleeding (%)	1.8 / 1.4	n.a.	1.7 / 0.8	0.44 (0.02–11.13)	3.7 / 2.7	0.73 (0.11–3.01)
Major vascular complications (%)	10.7 / 5.8	0.51 (0.17–1.61)	1.7 / 1.5	0.88 (0.08–19.09)	3.7 / 2.7	0.73 (0.11–3.01)
Hospitalization (%)	1.8 / 0.7	0.40 (0.02–10.27)	1.7 / 0	n.a.	3.2 / 4.1	1.28 (0.27–4.74)
Valve dysfunction (%)	0 / 0	n.a.	0 / 0	n.a.	1.4 / 0	n.a.
NYHA Class III or IV (%)	5.04 / 8.1	1.55 (0.46–7.08)	1.7 / 2.3	1.33 (0.17–27.11)	80.6 / 60.3	0.36 (0.21–0.64)
CCS III or IV (%)	0 / 0	n.a.	0 / 0	n.a.	2.5 / 6.4	2.62 (0.52–11.08)

Values are median (interquartile range) unless indicated otherwise

*BAV* Balloon aortic valvuloplasty, *CCS* Canadian cardiovascular society, *MI* Myocardial infarction, *NYHA* New York Heart Association, *n.a.* Not applicable, *PPI* Permanent pacemaker implantation, *TA* Transapical, *TAo* Transaortic, *TF* Transfemoral, *X*_*w*_ Transcatheter aortic valve implantation with balloon aortic valvuloplasty, *X*_*wo*_ *= TAVI* Transcatheter aortic valve implantation without balloon aortic valvuloplasty

No significant differences in 30-day outcomes between the no-BAV and BAV groups were seen in the analysis by route of access (Table 6) except for a reduced likelihood of being in NYHA Class III/IV in the no-BAV group treated with TAo-TAVI (60.3% versus 80.6%; OR 0.36, 95% CI 0.21–0.64).

## Discussion

This meta-analysis of three prospective multicenter registry studies confirmed that omission of the BAV pre-dilation step prior to TAVI using the balloon-expandable Edwards SAPIEN 3 (or XT) transcatheter heart valve had no adverse effect on procedural or 30-day and 6 months outcomes. On the contrary, it was associated with shorter procedure times and less PVL. The results suggest that BAV is unnecessary in the majority of patients undergoing TF-TAVI, TA-TAVI or TAo-TAVI.

### Patient and disease characteristics

Patients in the BAV group were more likely to have peripheral artery disease whereas those in the no-BAV group were more likely to have undergone previous cardiovascular interventions and had a higher EUROSCORE II score. Specific aortic valve-related disease characteristics were generally similar between the groups. The reasons that clinicians selected conventional or direct TAVI were not evaluated in the meta-analysis. One of the individual studies reported that common reasons for omitting BAV were a desire to reduce procedural duration and a perceived risk of cerebral microemboli. Common reasons for performing BAV included to facilitate crossing the native aortic valve and doubts about the choice of valve size [17] (Schymik G, Rudolph TK, Jacobshagen C, Rothe J, Treede H, Kerber S, Frank D, Sykorova L, Okamoto M, Thoenes M, et al. Balloon-expandable transfemoral transcatheter aortic valve implantation with or without pre-dilation – findings from the EASE-IT TF multicentre registry, submitted).

### Periprocedural data

As would be expected, omitting the BAV step led to a significantly shorter total procedural time (by approximately 20 min compared with the procedure including BAV). Fluoroscopy time was also shorter in the no-BAV group (by 5 min), although the quantity of contrast agent used was not reduced significantly. A previous meta-analysis of clinical studies also found that procedural time was reduced by approximately 20 min with the omission of BAV; that analysis found no difference in fluoroscopy time but did report reduced use of contrast medium [12].

It might be expected that there would be a potentially greater need for post-procedural dilation in the group that did not undergo pre-dilation, as seen in one previous registry study where the rate was 26% in the no-BAV group compared with 6% in the BAV group [10]. However, in the current meta-analysis post-delivery balloon dilation was less common in the no-BAV group than in the BAV group, driven largely by TF-TAVI patients. Other meta-analyses of clinical studies found no significant difference in post-procedural dilation between BAV and non-BAV groups [11, 12].

The rate of device success in the current meta-analysis was high regardless of whether or not BAV was performed, and did not differ significantly between the groups, which is consistent with other registry studies [9, 14] and meta-analyses of clinical studies [11, 12]. However, the adjusted analysis in the current meta-analysis suggested that omission of BAV was associated with a reduced risk of device malfunction. Overall, these results suggest that decisions not to perform BAV were appropriate for patients actually selected for this approach. Of note, the percentage of TAVR procedures without BAV increased over time in the three analyzed studies which might be mainly attributed to the increasing experience of operators and growing evidence that omitting BAV is not disadvantageous in the overall TAVR procedure.

### Periprocedural complications

Patients undergoing TAVI and/or BAV can develop arrhythmias, coronary obstruction or severe aortic regurgitation which may necessitate the use of catecholamines. In the current meta-analysis, the need for catecholamine use was reduced when BAV was omitted. In addition, the risk of atrioventricular block was lower when BAV was omitted, although this relationship was no longer significant when the analysis was fully adjusted. Moderate/severe paravalvular regurgitation after TAVI was uncommon in either group, but it appeared to be more common in the BAV group than the no-BAV group among those who underwent TAo-TAVI.

### Early safety/efficacy

The current meta-analysis found no significant differences between the BAV and no-BAV groups in terms of the risk of mortality, stroke, permanent pacemaker implantation or other clinical outcomes at 30 days after TAVI, with one exception. TAVI without BAV was associated with a reduced risk of the patient being in NYHA class III/IV at day 30, which remained significant in the adjusted analysis. The proportion of patients in NYHA class III/IV at baseline did not differ between the groups. The reason for this finding is not clear, but it may relate to the fact that in the initially conducted studies (ROUTE and EASE-it TA) patients were mainly treated with BAV whereas in the latter trial (EASE-it TF) the majority of patients underwent TAVR without BAV. When analyzing these studies together the access route might be the main driver for worse outcome regarding NYHA class, since non-TF treated patients would be expected to undergo a longer recovery period. Other meta-analyses of clinical studies have not reported differences in NYHA class at day 30 [11, 12].

### Limitations

The non-randomized nature of the registry studies makes them susceptible to bias. The decision about whether or not to perform BAV was made by the treating clinician and may have been determined by the severity of illness, complexity of the valve anatomy and their perception of the likelihood of successful implantation; thus, it is possible that BAV might have been selected for more complex cases. The procedures being evaluated are subject to a learning curve and less experienced surgeons/teams might have been more likely to perform BAV. However, the meta-analysis was adjusted for potential confounders where possible. All three studies evaluated balloon-expandable Edwards SAPIEN valves (primarily the SAPIEN 3), which reduced confounding associated with the use of different devices; however, the results therefore do not necessarily apply to TAVI performed using other transcatheter valve systems. When analyzed by route of access, there were too few 30-day outcome events to allow a meaningful interpretation.

## Conclusions

This meta-analysis of real-world data indicates that TAVI performed without BAV is advantageous as it has a high device success rate, reduced procedure and fluoroscopy time, reduced risk of PVL and no adverse effect on short-term clinical outcomes.

## Data Availability

Available from the corresponding author upon reasonable request.

## Abbreviations

aOR: Adjusted odds ratio
BAV: Balloon aortic valvuloplasty
OR: Odds ratio
PV: ParaValvular
TA: TransApical
TAo: TransAortic
TAVI: Transcatheter aortic valve implantation
TF: TransFemoral
